# Bayesian multitrait kernel methods improve multienvironment genome-based prediction

**DOI:** 10.1093/g3journal/jkab406

**Published:** 2021-11-29

**Authors:** Osval Antonio Montesinos-López, José Cricelio Montesinos-López, Abelardo Montesinos-López, Juan Manuel Ramírez-Alcaraz, Jesse Poland, Ravi Singh, Susanne Dreisigacker, Leonardo Crespo, Sushismita Mondal, Velu Govidan, Philomin Juliana, Julio Huerta Espino, Sandesh Shrestha, Rajeev K Varshney, José Crossa

**Affiliations:** 1 Facultad de Telemática, Universidad de Colima, Colima 28040, Mexico; 2 Departamento de Estadística, Centro de Investigación en Matemáticas, Guanajuato 36023, Mexico; 3 Departamento de Matemáticas, Centro Universitario de Ciencias Exactas e Ingenierías (CUCEI), Guadalajara 44430, Mexico; 4 Department of Agronomy, Kansas State University, 2004 Throckmorton Plant Science Center, Manhattan, KS 66506, USA; 5 International Maize and Wheat Improvement Center (CIMMYT), Km 45, Carretera Mexico-Veracruz, CP 52640, Texoco, Edo. de Mexico, Mexico; 6 Campo Experimental Valle de Mexico, Instituto Nacional de Investigaciones Forestales, Agricolas y Pecuarias (INIFAP), Universidad Autónoma de Chapingo, Texcoco 56235, Mexico; 7 International Crops Research Institute for the Semi-Arid Tropics (ICRISAT), Hyderabad 502324, India; 8 State Agricultural Biotechnology Centre, Centre for Crop and Food Innovation, Food Futures Institute, Murdoch University, Murdoch 6150, Australia; 9 Colegio de Postgraduados, Montecillos, Edo. de México 56230, Mexico

**Keywords:** multitrait, kernel methods, plant breeding, genomic-enabled prediction, genomic prediction, GenPred, shared data resources

## Abstract

When multitrait data are available, the preferred models are those that are able to account for correlations between phenotypic traits because when the degree of correlation is moderate or large, this increases the genomic prediction accuracy. For this reason, in this article, we explore Bayesian multitrait kernel methods for genomic prediction and we illustrate the power of these models with three-real datasets. The kernels under study were the linear, Gaussian, polynomial, and sigmoid kernels; they were compared with the conventional Ridge regression and GBLUP multitrait models. The results show that, in general, the Gaussian kernel method outperformed conventional Bayesian Ridge and GBLUP multitrait linear models by 2.2–17.45% (datasets 1–3) in terms of prediction performance based on the mean square error of prediction. This improvement in terms of prediction performance of the Bayesian multitrait kernel method can be attributed to the fact that the proposed model is able to capture nonlinear patterns more efficiently than linear multitrait models. However, not all kernels perform well in the datasets used for evaluation, which is why more than one kernel should be evaluated to be able to choose the best kernel.

## Introduction

Genomic selection (GS) has been widely adopted because its predictive methodology enables the selection of candidates before phenotypes are available on all individuals (Meuwissen *et al.* 2001). Current research in GS includes the use of prediction models in GS that were successful in other fields, or the adaptation or development of specific models for GS (Montesinos-López *et al.* 2019b, 2019c), and models that couple mechanistic and statistical approaches (Tong *et al.* 2020). At the same time, breeders usually select multiple traits that are often genetically correlated, with correlations ranging from weak to strong. Often analyses of multitrait data are performed with uni-trait (UT) models, which assume zero genetic and residual covariances among these traits so that information from other traits is not used (Montesinos-López *et al.* 2019b) when obtaining expected breeding values of the evaluated individuals for the traits under study (Okeke *et al.* 2017). However, the optimal estimation process is composed of the combination of information from multiple traits and estimated breeding values using the multitrait (MT) models (van der Werf 1992; Ducrocq 1994; Okeke *et al.* 2017). 

The use of UT models is very common, partly due to the lower number of existing MT models. However, the attraction of MT models continues growing, as pointed out by Mbebi *et al.* (2021). UT models are trained using only one dependent variable. However, these models are unable to capture the correlation between traits when only one dependent variable is used, that is, when the training process is done separately for each trait (Montesinos-López *et al.* 2019b), whereas MT models are trained using all the available traits simultaneously, which is why they are able to capture the correlation between traits. When this correlation between traits is moderate or large, most of the time the prediction performance of MT models is better than that of UT models (Montesinos-López *et al.* 2016, 2019b, 2019c, 2020).

In MT models, even when the traits are unfavorably correlated (opposite signs), improvement of the prediction performance is expected as compared to UT models because the borrowing of information is possible (Neyhart *et al.* 2019). However, from a practical perspective, unfavorable correlations are common and complicate breeders’ decisions (Neyhart *et al.* 2019). Opposite directions of such correlations imply an unfavorable response in one trait when selecting on another (Falconer and Mackay 1996); thus the underlying cause will impact the prospects of long-term improvement (Neyhart *et al.* 2019).

There is empirical evidence that MT models (frequentist and Bayesian) outperform UT when the traits are correlated, as reported by some authors such as Calus and Veerkamp (2011), Jia and Jannink (2012), Jiang *et al.* (2015), Montesinos-López *et al.* (2016), He *et al.* (2016), and Schulthess *et al.* (2018), who reported that, at least for some traits, MT models outperform UT models in terms of prediction accuracy. Schulthess *et al.* (2018) also reported that, compared to UT models, MT models improve parameter estimates. Small differences are observed between frequentist and Bayesian methods in terms of prediction performance.

However, it has also been reported that when the correlation between traits is low, MT models are not really advantageous (Montesinos-López *et al.* 2016, 2018, 2019), since MT models provide less benefits when the degree of relatedness between traits is low (Montesinos-López *et al.* 2016, 2018, 2019). An early study of multivariate genomic prediction (Jia and Jannink 2012) showed the usefulness of multivariate models, but large differences were only observed when variable selection methods (BayesA and BayesC) were applied to nonpolygenic traits (20 QTLs), and little difference was observed in polygenic traits.

The following seven advantages of MT models with regard to UT models have been pointed out by Montesinos-López *et al.* (2019b): (1) MT models represent complex relationships between traits more efficiently; (2) they exploit not only the correlation between lines, but also the correlation between traits; (3) they are much more interpretable than a series of UT models; (4) they are more computationally efficient (less time for training) than multiple UT models individually; (5) they improve the selection index because they allow more precise estimates of random effects of lines and genetic correlation between traits; (6) they can improve indirect selection because they increase the precision of genetic correlation parameter estimates between traits; and (7) they improve the power of hypothesis testing better than UT models.

Although MT models have many advantages over UT models, they require the estimation of more parameters (*i.e.*, genetic and error covariances), which affects the prediction performance of the MT models as well as the accuracy of breeding value estimates. The larger the number of traits, the larger the required number of parameters that need to be estimated (Runcie *et al.* 2021). Also, the more complex the model is and the larger the number of traits included, the greater chances there are of facing convergence problems in the analysis (Runcie *et al.* 2021). This means that MT models require more data to be able to accurately estimate the additional parameters (Okeke *et al.* 2017). The optimum training size depends upon the effective population size and the available genetic diversity within the population (Arojju *et al.* 2020). In general, results have shown that Bayesian MT methods have less issues related to convergence problems than frequentist MT methods (Montesinos-López *et al.* 2019b).

However, despite these seven advantages of MT models, most of them are unable to capture complex nonlinear patterns of the inputs. For example, MT models with a linear predictor are unable to capture these complex nonlinear patterns (Cuevas *et al.* 2016, 2017); however, it is quite straightforward to use the machinery of linear models for nonlinear tasks using Reproducing Kernel Hilbert Spaces (RKHS) methods (Gianola and van Kaam 2008). The use of RKHS methods for UT analysis is very common in GS (Cuevas *et al.* 2016, 2017; Crawford *et al.* 2018). For example, Long *et al.* (2010) reported that RKHS methods outperformed linear models in body weight of broiler chickens. Crossa *et al.* (2010) reported better prediction performance of RKHS methods with regard to linear Bayesian Lasso regression in wheat. In maize and wheat data, Cuevas *et al.* (2016, 2017, 2018, 2020) reported a greater performance of RKHS with Gaussian kernels over linear GBLUP for several UT genomic predictions incorporating genomic × environment interaction. Cuevas *et al.* (2019) also reported that nonlinear kernel methods (Gaussian kernel and arc-cosine kernel) outperformed linear kernel methods in terms of prediction performance using markers and near infrared spectroscopy data in the predictor pedigree.

The basic idea of RKHS methods is to project the original independent variables given in a finite dimensional vector space into an infinite-dimensional Hilbert space (Gianola and van Kaam 2008). Kernel methods transform the independent variables (inputs) using a kernel function, and then the transformed inputs can be used in conventional machine learning techniques at a low computational cost and repeatedly, with better results in terms of prediction performance (Shawe-Taylor and Cristianini 2004). RKHS methods based on implicit transformations have become very popular in analyses of nonlinear patterns in datasets from various fields of study. Kernel methods obtain measures of similarity between objects that do not have natural vector representation (Montesinos-López *et al.* 2021).

Due to its many attractive characteristics, the mixed-model framework under a frequentist approach is still very popular in GS for the implementation of MT models. However, the adoption of the Bayesian paradigm in plant breeding continues to grow due to the great computational advancements and new methodological applications and elucidations. Bayesian MT models offer some of the following advantages mentioned by Montesinos-López *et al.* (2019b): (1) they allow prior information to be incorporated; (2) they do not need good starting values to estimate parameters of interest such as the restricted maximum likelihood; (3) they increase the precision of parameter estimates (smaller standard errors); (4) conclusions can be drawn about the correlations between the dependent variables, notably, the extent to which the correlations depend on the individual and on the group level; (5) testing whether the effect of an explanatory variable on dependent variable Y1 is larger than its effect on Y2, when Y1 and Y2 data were observed (totally or partially) in the same individuals, is possible only by means of a multivariate analysis; (6) when attempting to carry out a single test of the joint effect of an explanatory variable on several dependent variables, a multivariate analysis is also required; such a single test can be useful, *e.g.*, to avoid the danger of chance capitalization, which is inherent to carry out a separate test for each dependent variable; and (7) it does not have strong identifiability problems. In general, the MT Bayesian approach has the advantage of being more parsimonious and providing a more informative and powerful analysis. However, Bayesian MT analysis is computationally more demanding than univariate analysis, and its implementation is therefore many times impractical.

Furthermore, the implementation of conventional MT (frequentist and Bayesian) models is, in general, computationally demanding (Runcie *et al.* 2021). The fragility of these methods is due to the number of variance–covariance parameters that must be estimated, which increases quadratically with the number of traits (Runcie *et al.* 2021). The computational demands increase even more dramatically, from cubically to quantically, with the number of traits (Zhou and Stephens 2014) because most algorithms require repeated inversion of large covariance matrices. These matrix operations dominate the time required to fit conventional MT models, leading to models that take days, weeks, or even years to converge (Runcie *et al.* 2021).

In this study, we propose Bayesian kernel methods for the multitrait genome-enabled prediction of multienvironment trials. We applied the proposed methods to three extensive wheat multitrait multienvironment trial datasets and compared the prediction performance using four kernels—linear (GBLUP), Gaussian kernel (GK), polynomial kernel (PK) and sigmoid kernel (SK)—and conventional Bayesian multitrait Ridge Regression (BRR) under two scenarios: Scenario 1, in which all traits are missing in the testing set (MT), and Scenario 2, in which only a fraction of the traits are missing in the testing set (MT_P). We also evaluated the prediction performance with and without including genotype× environment interaction (G × E) under a multitrait framework. Finally, we also provide the R code to implement these methods in conventional Bayesian multitrait software.

## Materials and methods

### Bayesian multitrait kernel model

This model is given in (1) as:

(1) $$Y=1_{n}\mu^{T}+X_{E}\beta_{E}+Z_{L}g+Z_{EL}gE+\epsilon$$

where Y is the matrix of phenotypic response variables of order $n\times n_{T}$; with n=JI and J and I denotes the number of lines and environments respectively. Y is ordered first by environments and then by lines, $n_{T}$ denotes the number of traits, $1_{n}$ is a vector of ones of length n, $\mu^{T}$ is a vector of intercepts for each trait of length $n_{T}$, T denotes the transpose of a vector or matrix, that is, $\mu ={\left[\mu_{1},\;\ldots ,\;\mu_{n_{T}}\right]}^{T},\;{\;X}_{E}$ is the design matrix of environments of order n×I, $\beta_{E}$ is the matrix of beta coefficients for environments with a dimension of $I\times n_{T}$, $Z_{L}$ is the design matrix of lines of order n×J, g is the matrix of random effects of lines of order $J\times n_{T}$ distributed as $g∼{MN}_{J{\times n}_{T}}\left(0,K^{l},\Sigma_{T}\right)$, that is, with a matrix-variate normal distribution with parameters M=0, $U=K^{l}$, and $V=\Sigma_{T}$, $K^{l}$ is the lth type of kernel matrix built with marker data (equivalent to a genomic relationship matrix) of order J×J that captures linear or nonlinear relationships (l=linear, Gaussian, polynomial and sigmoid) and $\Sigma_{T}$ is the variance–covariance matrix of traits of order $n_{T}\times n_{T}$.

Note that $Z_{L}g$ are the BLUPs of lines of the $n_{T}$ traits, but repeated in the I environments. $Z_{\mathrm{EL}}$ is the design matrix of the genotype × environment interaction of order n×JI, gE is the matrix of genotype × environment interaction random effects distributed as $\mathrm{gE}∼{MN}_{J{I\times n}_{T}}\left(0,K^{l}\;⊗\Sigma_{E},\Sigma_{T}\right)$, where $\Sigma_{E}$ is a diagonal variance–covariance matrix of environments of order I×I, and $K^{l}\;⊗\Sigma_{E}$ is the Kronecker product of the lth type of kernel matrix of lines and the environmental relationship matrix. Furthermore, the term $Z_{\mathrm{EL}}gE$ contains the BLUPs corresponding to the genotype × environment interaction terms of the $n_{T}$ traits. ϵ is the residual matrix of dimension$\;n\times n_{T}$ distributed as $\epsilon ∼{MN}_{n{\times n}_{T}}\left(0,I_{\mathrm{IJ}},R\right)$, where R is the residual variance–covariance matrix of order $n_{T}\times n_{T}$. The criteria for using these four kernels (linear, Gaussian, polynomialand sigmoid) were that these are very popular kernels used in statistical science and two of them in genomic prediction (linear and Gaussian).

### The kernel methods

The *linear kernel* (LK) was computed as $K\left(x_{i},x_{j}\right)=x_{i}^{T}x_{j}$ (Shawe-Taylor and Cristianini 2004), since $x_{i}^{T}$ and $x_{j}^{T}$ are any two rows of the scaled matrix of markers (X of order J×p) divided by the square root of the total number of markers (p) then this is indeed the linear kernel relationship matrix proposed by Van Raden (2008) and called Genomic Best Linear Unbiased Predictor (GBLUP). The *polynomial kernel* (PK) was computed as $K\left(x_{i},x_{j}\right)={\left({\gamma x}_{i}^{T}x_{j}+a\right)}^{d}$, where a=0 is a real scalar, γ =1 and d=3 is a positive integer (Shawe-Taylor and Cristianini 2004). The *sigmoidal kernel* (SK) was computed as $K\left(x_{i},x_{j}\right)=\tan h\left(x_{i}^{T}x_{j}+a\right)$, where tanh is the hyperbolic tangent defined as $\tan h(z)\;=\;\sin h(z)/\cos h(z)=\frac{\exp\left(z\right)-\exp(-z)}{\exp\left(z\right)+\exp(-z)}$ (Shawe-Taylor and Cristianini 2004). The *Gaussian kernel* (GK), also known as the radial basis function kernel, was computed as $K\left(x_{i},x_{j}\right)=e^{-\gamma {\left\|x_{i}-x_{j}\right\|}^{2}\;}=e^{-\gamma [x_{i}^{T}x_{i}-2x_{i}^{T}x_{i}+x_{j}^{T}x_{j}]\;}$, where γ is a positive real scalar (Shawe-Taylor and Cristianini 2004) and in this application, the parameter γ used was γ=1, assuming that the markers were scaled.

### Computational implementation of the Bayesian multitrait kernel model

Note that when $\Sigma_{T}$, $\Sigma_{E}$, and R are diagonal matrices, model (1) is equivalent to separately fitting a univariate linear model to each trait. Also, when a linear kernel for $K^{l}$ is used in model (1), the model is equivalent to a conventional multitrait GBLUP model. The Bayesian multitrait kernel model (1) can be implemented in the BGLR package of de los Campos and Pérez-Rodríguez (2014). The github version of the BGLR R library can be accessed at https://github.com/gdlc/BGLR-R and can be installed directly in the R console by running the following commands: *install.packages(‘devtools’); library(devtools); install_github* (https://github.com/gdlc/BGLR-R)*.* First we need to have computed: $X_{E}$ denotes the design matrix of environments, $Z_{L}$ denotes the design matrix of lines, $K^{l}$ any of the 4 kernels described above (l=linear, Gaussian, polynomial andsigmoid), $\mathrm{KL}=Z_{L}K^{l}Z_{L}^{T}$, $\mathrm{KE}=X_{E}X_{E}^{T}$, and KLE=KL°KE (see Appendix B).

This implementation of model (1) can be carried out with this version of the BGLR package as follows:

ETA = list(Env = list (X = XE, model='FIXED'), Line=list (K= KL, model=’ RKHS’), LinexEnv= list (K= KLE, model=’ RKHS’))

A = Multitrait(y = Y, ETA=ETA, resCov = list (type = ′UN', S0 = SR, df0 = vR), nIter = nI, burnIn = nb)

The first argument in the multitrait function is the response variable that is a phenotype matrix, in which each row corresponds to the measurements of $n_{T}$ traits in each individual. The second argument is a list predictor in which the first sub-list specifies the design matrix and prior model to the fixed effects part of the predictor in model (1), while the second sub-list specifies the parameters of the distribution of random genetic effects (g), where the KL is the expanded genomic relationship matrix specified, and which accounts for the similarity between individuals based on marker information. The third sub-list specifies the parameters of the distribution of random genotype by environment effects of gE, where the **KLE** is the genomic relationship matrix specified, and which accounts for the similarity between individuals. df0 = $v_{T}$ and S0 = $S_{T}$ are the degrees of freedom parameter ($v_{T}$) and the scale matrix parameter ($S_{T}$) of the inverse Wishart prior distribution for $\Sigma_{T}$, respectively. In the third argument (resCOV), S0 and df0 are the Scale matrix parameter ($S_{R}$) and the degree of freedom parameter ($v_{R}$) of the inverse Wishart prior distribution for R. The last two arguments are the required number of iterations (**nI**) and the burn-in period (**nb**) to run the Gibbs sampler.

### Datasets 1–3: elite wheat yield trial years 2013–2014, 2014–2015, and 2015–2016

These three datasets were collected by the Global Wheat Program (GWP) of the International Maize and Wheat Improvement Center (CIMMYT) and belong to elite yield trials (EYT) established in four different cropping seasons with four or five environments each. The lines involved in each of the environments of the same year are the same, but those in different years are different lines. EYT dataset 1 was sown in 2013–2014 and contains 767 lines, EYT dataset 2 was established in 2014–2015 and contains 775 lines and EYT dataset 3 was cultivated in 2015–2016 and contains 964 lines. The experimental design used was an alpha-lattice design and the lines were sown in 39 trials, each covering 28 lines and two checks in six blocks with three replications. In each dataset, several traits were available for some environments and lines. In this study we included four traits that were measured for each line in each environment: days to heading (DTHD, number of days from germination to 50% spike emergence), days to maturity (DTMT, number of days from germination to 50% physiological maturity or the loss of the green color in 50% of the spikes), plant height, and grain yield (GY). Full details of the experimental design and how the BLUEs were computed are given in Juliana *et al.* (2018).

In EYT 2013–2014 dataset 1, the lines under study were evaluated in 4 environments, while in EYT 2014–2015 dataset 2 and EYT 2015–2016 dataset 3, the lines were evaluated in five environments. For EYT dataset 1, the environments were bed planting with five irrigations (Bed5IR), flat planting and five irrigations (Flat5IR), early heat (EHT), and late heat (LHT). For EYT dataset 2, the environments were bed planting with two irrigation levels (Bed2IR), bed planting with five irrigations levels (Bed5IR), flat planting with five irrigation levels (Flat5IR), early heat (EHT) and late heat (LHT). Finally, for EYT dataset 3, the environments were bed planting with two irrigation levels (Bed2IR), bed planting with five irrigations levels (Bed5IR), flat planting with five irrigation levels (Flat5IR), flat planting with drip irrigation (FlatDrip), and late heat (LHT).

Genome-wide markers for the 2506 (667 + 775 + 964) lines in the three datasets were obtained using genotyping-by-sequencing (GBS; Elshire *et al.* 2011; Poland *et al.* 2012) at Kansas State University using an Illumina HiSeq2500. After filtering, 2038 markers were obtained from an initial set of 34,900 markers. The imputation of missing markers data was carried out using LinkImpute (Money *et al.* 2015) and implemented in TASSEL (Bradbury *et al.* 2007), version 5. Lines that had over 50% of missing data were removed and 2506 lines were used in this study (767 lines in the first dataset, 775 lines in the second dataset, and 964 lines in the third dataset). Also expected is a high level of relatedness given by pedigree or kinship between lines within a year of testing and also across years of testing due to the nature of the lines under study.

### Evaluation of prediction accuracy with random cross-validation

The prediction accuracy of the Bayesian multitrait kernel model was evaluated with cross-validation (CV). A fivefold CV was implemented and the original dataset was partitioned into five subsamples of equal size, and each time, four of them were used for training and the remaining one for testing. In fivefold CV, one observation cannot appear in more than onefold. In the design, some lines can be evaluated in some, but not all, target environments, which mimics a prediction problem faced by breeders in incomplete field trials. Our validation strategy is exactly the same as the strategy denoted as CV2 that was proposed and implemented by Jarquín *et al.* (2014), in which a certain portion of test lines (genotypes) in a certain portion of test environments is predicted, since some test lines that were evaluated in some test environments are assumed to be missing in others.

We used the mean square error of prediction [$\mathrm{MSE}=\frac{1}{T}(\sum_{i=1}^{T}{\left(y_{i}-\hat{f}(x_{i})\right)}^{2}$, where $y_{i}$ is the observed value of the *i*th observation, $\hat{f}(x_{i})$ is the prediction that $\hat{f}$ gives to the *i*th observation and T is the number of observations in the testing set] to evaluate the prediction performance, since we are working with continuous variables and MSE was calculated from each environment in each trait for each of the testing sets. The formula given above was used to compute the MSE error in each fold, but the average of all folds was reported as a measure of genome-based prediction performance. The lower the average of MSE, the better the prediction performance. All the analyses were carried out using the R statistical software (R Core Team 2020).

## Results

The results are given in two sections that correspond to datasets 1 and 2. In each dataset, the genome-based prediction performance was assessed without including G × E interactions and including G × E interactions. Both cases are provided under the following scenarios: (1) when all the traits in the testing set are predicted (standard MT method) and (2) when only a fraction of the traits in the testing sets are predicted (MT_P). Two traits were considered: DTHD and DTMT. For simplicity and clarity, results from dataset 3 are provided in Appendix A, where genome-based predictions measured under the MSE of prediction without G × E interaction and with G × E interactions are described under the two scenarios, MT and MT_P.

Results are presented for each trait including (I) and ignoring (WI) G × E interaction for each of the scenarios, MT and MT_P in the form of tables and figures for each environment (of each of the datasets) and across environments.

### Dataset 1 (EYT 2013–2014)

#### DTHD (without G × E interaction, WI)

**Figure 1:**
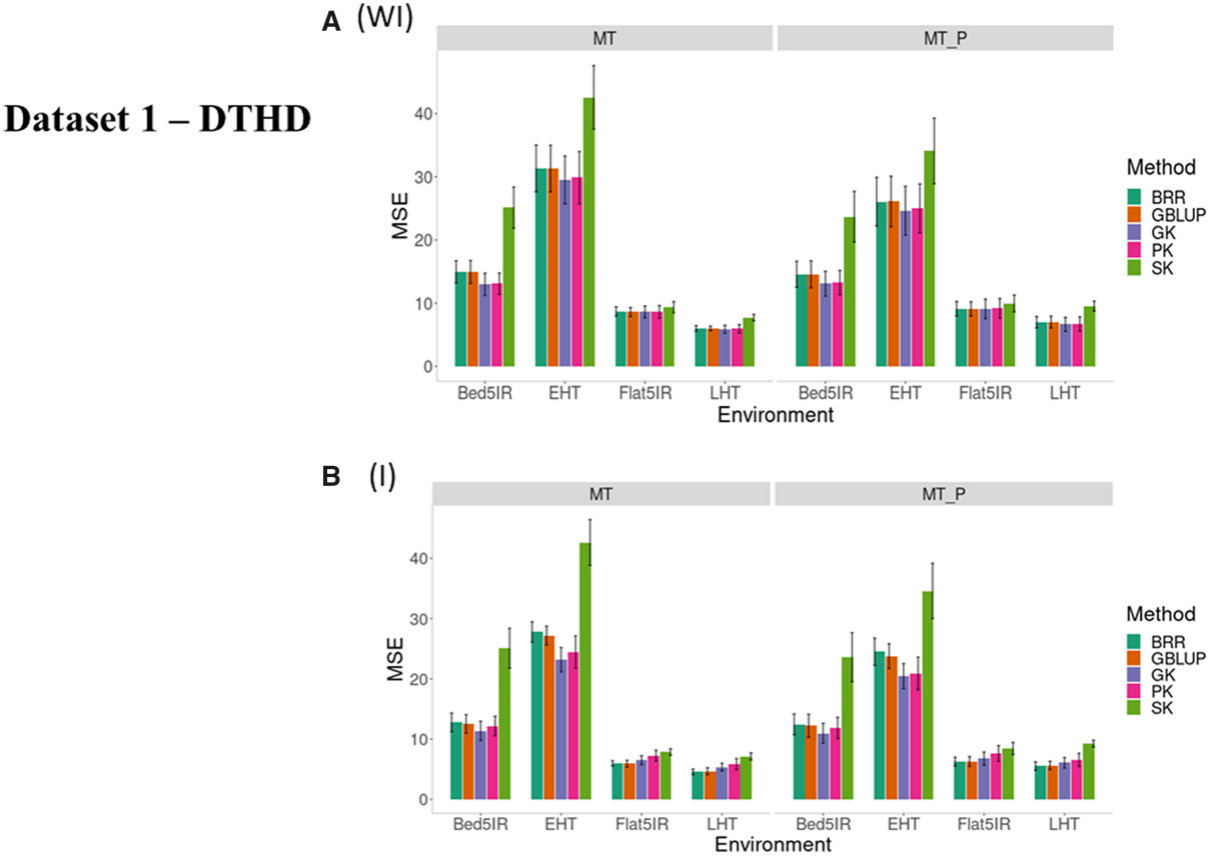
Dataset 1—DTHD. Prediction performance in terms of mean square error of prediction (MSE) for five methods (BRR, GBLUP, GK, PK, and SK) (A) without G × E interaction (WI) and (B) including G × E interaction (I) for four environments (Bed5IR, EHT, Flat5IR and LHT) and two scenarios (MT and MT_P).

We first compared the prediction performance for trait DTHD in terms of MSE for the methods (Figure  1A, WI, and Table  1) without G × E interaction under conventional multitrait Bayesian Ridge Regression (BRR) and four types of kernels [linear GBLUP, Gaussian (GK), polynomial (PK), and sigmoid (SK)] when all traits in the testing set are predicted (MT) and when only a fraction of the traits is predicted (MT_P). In Figure  1A, WI, and Table  1 under both scenarios (MT and MT_P), the best performance for most of the four environments was observed under the multitrait GK and the worst was found under the multitrait SK for both MT and MT_P scenarios. In environment EHT under scenario MT_P, the predictions were considerably better than under scenario MT, while in environment LHT, scenario MT was slightly better than scenario MT_P (Table  1 and Figure  1A, WI).

**Table 1:** Dataset 1 EYT 2013–2014

		Models and methods					Models and methods				
		BRR	GBLUP	GK	PK	SK	BRR	GBLUP	GK	PK	SK
			
Env.	Scenario	Without G × E (WI)					With G × E (I)				
		DTHD									
Bed5IR	MT	14.95	14.94	**12.96**	13.08	25.12	12.77	12.52	**11.36**	12.17	25.07
EHT	MT	31.32	31.30	**29.51**	29.86	42.57	27.77	27.19	**23.17**	24.43	42.62
Flat5IR	MT	8.68	**8.59**	8.61	8.62	9.36	5.97	**5.92**	6.49	7.26	7.85
LHT	MT	6.00	5.99	**5.87**	5.94	7.71	**4.56**	**4.68**	5.36	5.84	7.12
Bed5IR	MT_P	14.57	14.56	**13.07**	13.26	23.68	12.46	12.21	**10.97**	11.86	23.58
EHT	MT_P	26.06	26.09	**24.63**	24.98	34.09	24.50	23.75	**20.45**	20.89	34.58
Flat5IR	MT_P	9.12	**9.09**	**9.09**	9.18	9.96	**6.25**	**6.29**	6.75	7.62	8.45
LHT	MT_P	6.97	6.99	**6.63**	6.71	9.54	**5.52**	**5.63**	6.06	6.56	9.23
		DTMT									
Bed5IR	MT	11.62	11.58	**10.17**	10.18	18.88	10.25	9.94	**9.07**	9.37	18.93
EHT	MT	26.21	26.22	**24.72**	24.89	35.73	23.81	23.55	**19.81**	20.35	37.19
Flat5IR	MT	8.92	**8.88**	9.37	9.45	8.35	6.58	6.58	**7.58**	8.30	6.64
LHT	MT	7.80	7.77	**7.58**	7.62	10.83	6.52	6.45	**6.44**	6.93	10.77
Bed5IR	MT_P	11.47	11.49	**10.34**	10.43	17.96	10.21	9.91	**8.99**	9.40	18.05
EHT	MT_P	19.56	19.61	**18.38**	18.58	26.16	18.94	18.69	**15.41**	15.38	27.49
Flat5IR	MT_P	9.68	**9.66**	10.02	10.10	9.55	7.19	**7.16**	7.96	8.78	7.89
LHT	MT_P	8.42	8.42	**8.00**	8.11	11.83	7.24	**7.20**	7.30	7.69	12.13

Average mean squared error (MSE) of prediction for five multitrait multienvironment model-methods: BRR, Bayesian ridge regression; GBLUP, genomic best linear unbiased predictor; GK, Gaussian kernel; PK, polynomial kernel; SK, sigmoidal kernel without G × E (WI) and with G × E (I) for two scenarios (MT and MT_P) for four environments (Bed5IR, EHT, Flat5IR, LHT) and two traits (DTHD, days to heading and DTMT, days to maturity). Boldface indicates model-method with the lowest MSE for the environment.

**Figure 2:**
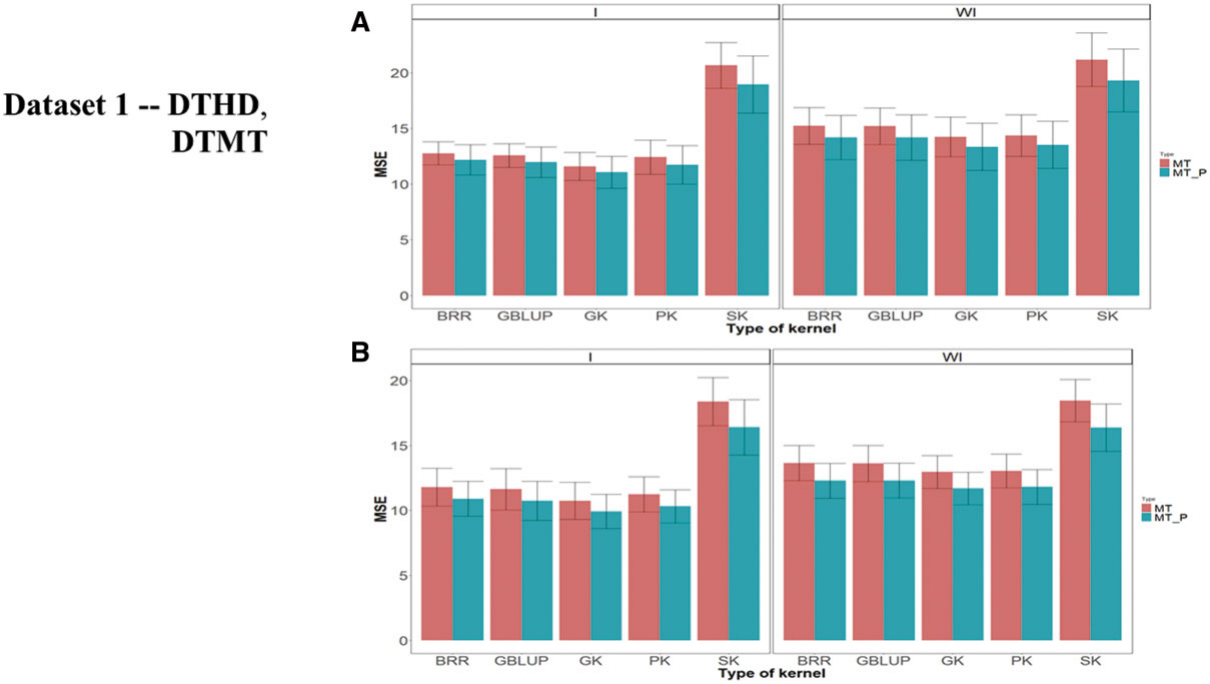
Dataset 1—DTHD and DTMT. Prediction performance across environments in terms of mean square error of prediction (MSE) for traits (A) DTHD with (I) and without (WI) including G × E interaction term for two scenarios (MP and MT_P) and (B) DTMT with (I) and without (WI) including G × E interaction term for two scenarios (MP and MT_P).

Across environments, multitrait GK was always better than the other kernels for MT and MT_P (Figure  2A, WI, and Table  2). For the MT predictions, the GK outperformed the BRR, GBLUP, PK and SK by 7.012%, 6.76%, 0.928%, and 48.8%, respectively, while across environments for the MT_P predictions, the GK outperformed the BRR, GBLUP, PK, and SK by 6.17%, 6.19%, 1.32%, and 44.64%, respectively. Under scenario 2, MT_P gave a slightly better genome-based prediction than under scenario MT.

**Table 2:** Dataset 1 EYT 2013–2014

	Models and methods					Models and methods				
	BRR	GBLUP	GK	PK	SK	BRR	GBLUP	GK	PK	SK
		
Scenario	Without G × E (WI)					With G × E (I)				
DTHD										
MT	15.24	15.20	**14.24**	14.37	21.19	12.77	12.58	**11.60**	12.43	20.67
MT_P	14.18	14.18	**13.36**	13.53	19.32	12.18	11.97	**11.06**	11.73	18.96
DTMT										
MT	13.64	13.61	**12.96**	13.03	18.44	11.79	11.63	**10.73**	11.24	18.38
MT_P	12.28	12.30	**11.68**	11.80	16.37	10.90	10.74	**9.92**	10.31	16.39

Average mean squared error (MSE) prediction across environments for five model-methods: BRR, Bayesian ridge regression; GBLUP, genomic best linear unbiased predictor; GK, Gaussian kernel; PK, polynomial kernel; SK, sigmoidal kernel without G × E (WI) and with G × E (I) for two scenarios (MT and MT_P), four environments (Bed5IR, EHT, Flat5IR, LHT), and two traits (DTHD, days to heading and DTMT, days to maturity). Boldface indicates model-method with the lowest MSE for each scenario.

#### DTHD (G × E interaction, I)

Taking into account the G × E interaction term, we also see that the worst performance was observed under the SK under both scenarios (MT and MT_P; Figure  1B, I, and Table  1). The best performance was observed under the GK under MT_P in environments Bed5IR and EHT, and BRR and GBLUP in environments Flat5IR and LHT. Large differences were not observed between the predictions without G × E interaction (Figure  1A, WI) and with G × E interaction (Figure  1B, I).

Across environments (Figure  2A, I, and Table  2) for MT predictions, the GK outperformed the BRR, GBLUP, PK, and SK by 10.35%, 8.47%, 7.15%, and 78.23%, respectively, while for scenario MT_P, the GK outperformed the BRR, GBLUP, PK, and SK by 10.18%, 8.25%, 6.08%, and 71.43%, respectively. There were increases in genome-based prediction when (1) including G × E (Figure  2A, I) compared to when ignoring G × E (Figure  2A, WI; Table  2) and (2) employing the MT_P scenario.

#### DTMT (without G × E, WI)

**Figure 3:**
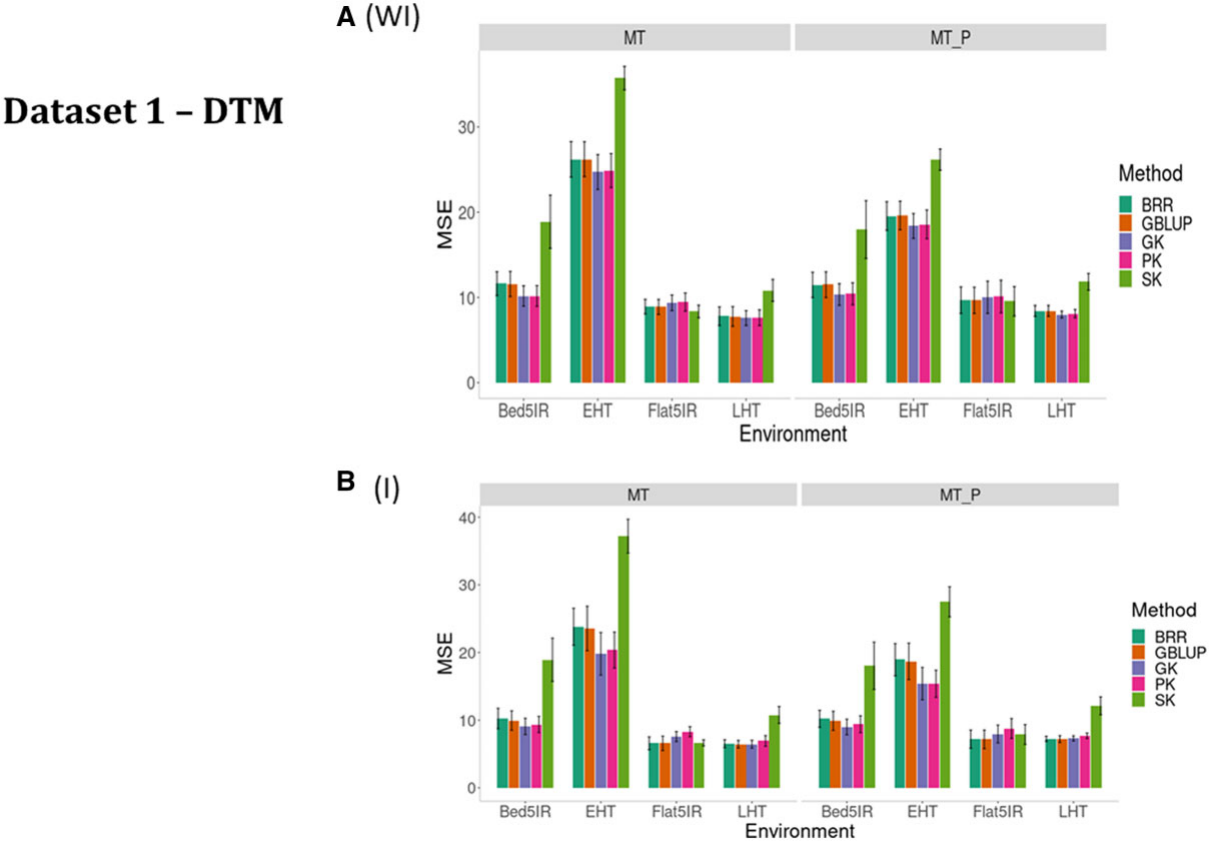
Dataset 1—DTMT. Prediction performance in terms of mean square error of prediction (MSE) for five methods BRR, GBLUP, GK, PK, and SK when (A) without G × E interaction (WI) and (B) including G × E interaction (I) for four environments (Bed5IR, EHT, Flat5IR, and LHT).

The prediction performance for trait DTMT is provided in terms of MSE for the five kernel methods (Figure  3A, WI, and Table  1) under conventional multitrait Ridge regression (BRR) and four types of kernels (GBLUP, GK, PK, and SK) under the same two scenarios (MT and MT_P). In Figure  3A, WI, and Table  1, it is observed that ignoring the G × E interaction term, under both scenarios (MT and MT_P), that the worst performance was for SK, while the best performance was the GK method for all environments except MT_P in Flat5IR (MSE = 9.66). The SK was considerably worse than the other methods under both scenarios (Figure  3A, WI). In environment LHT, scenario MT was slightly better than MT_P (Figure  3A, WI, and Table  1).

Across environments, under scenario MT predictions, the GK was better than BRR, GBLUP, PK, and SK by 5.23, 5.06, 0.57 and 42.34%, respectively, while under MT_P predictions, the GK outperformed the BRR, GBLUP, PK, and SK by 5.10, 5.23, 1.02 and 40.14%, respectively (Figure  2B, WI, and Table  2). The genome-based predictions under MT_P were better than under MT (Figure  2B, WI, and Table  2).

#### DTMT (G × E, I)

Considering the G × E interaction term, we also see that the worst performance was observed under the SK under both scenarios (MT and MT_P; Figure  3B, I, and Table  1). The best performance was observed under the GK in environments Bed5IR and EHT, and under BRR and GBLUP in environments Flat5IR and LHT. Large differences were not observed between the predictions without G × E interaction (Figure  3A, WI) and with G × E interaction (Figure  3B, I).

For trait DTMT across environment analyses, taking the G × E interaction into account, under MT and MT_P, the worst performance was observed under the SK, and in general, scenario MT_P was better than MT (Figure  2B, I, and Table  2). Under MT predictions across environments, the GK was superior in genomic-enabled prediction accuracy than BRR, GBLUP, PK, and SK by 9.90%, 8.43%, 4.76%, and 71.37%, respectively, whereas for MT_P, the GK was better than BRR, GBLUP, PK and SK by 9.98%, 8.31%, 3.97%, and 65.25%, respectively (Figure  2B, I, and Table  2). As for trait DHTD, there was a slight consistent increase in genome-based prediction accuracy when including G × E (Figure  2B, I) compared to when ignoring G × E (Figure  2B, WI) and for scenario 2 MT_P over scenario MT (Table  2).

### Summary of results for dataset 1

The nonlinear multitrait Gaussian kernel showed the best genome-based prediction accuracies in most of the environments for both traits, DTHD and DTMT, whereas the sigmoidal kernel (SK) gave the worst prediction. Consistently for the 4 kernel methods linear GBLUP, GK, PK, and SK, the model including G × E gave lower MSE than models ignoring G × E, whereas the scenario that included all the traits (MT) gave a slightly worse prediction accuracy than the scenario including only a fraction of the traits in the testing sets to be predicted (MT_P). Although these patterns are expressed in most (but not all) of the environments, the across environments analyses of Table  2 and Figure  2 clearly displayed these conclusions.

### Dataset 2 (EYT 2014–2015)

#### DTHD (without G × E, WI)

**Figure 4:**
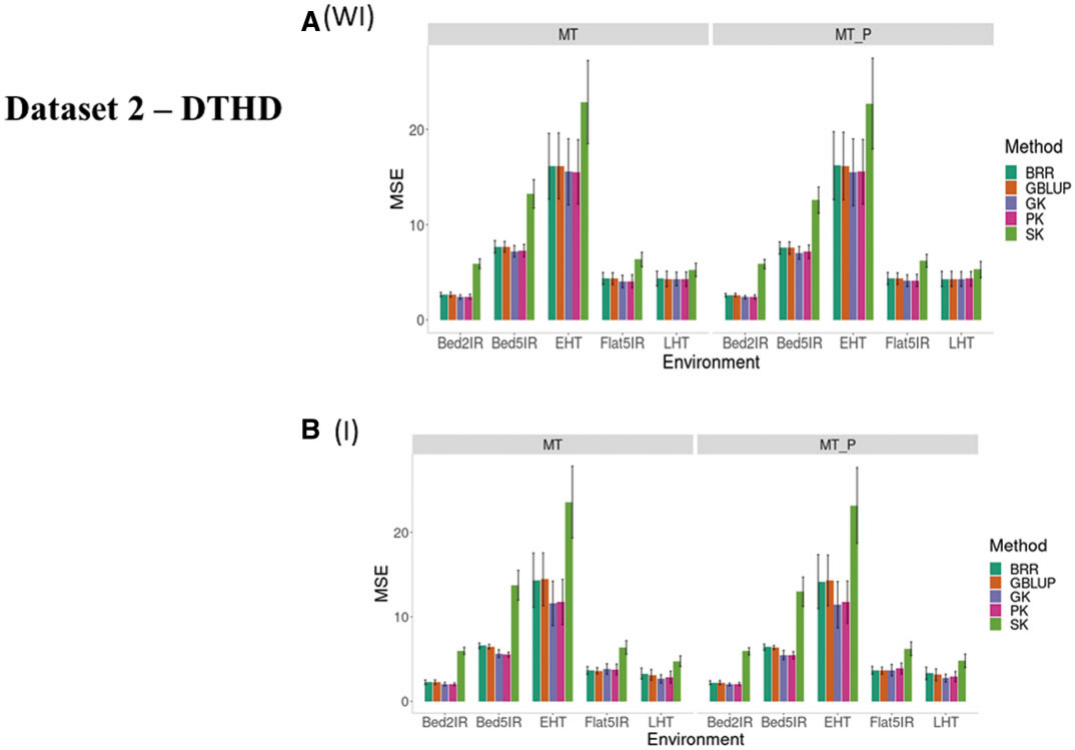
Dataset 2—DTHD. Prediction performance in terms of mean square error of prediction (MSE) for five methods (BRR, GBLUP, GK, PK, and SK) (A) without G × E interaction (WI) and (B) including G × E interaction (I) for five environments (Bed2IR, Bed5IR, EHT, Flat5IR, and LHT) and two scenarios (MT and MT_P).

We first compared the prediction performance of the five methods (Figure  4A, WI, and Table  3) under MT and MT_P scenarios when ignoring G × E (WI). The best performance was observed under the GK, and the worst, under the SK. The SK was also considerably worse than the other methods under both MT and MT_P (Figure  4A, WI). Figure  4A, WI, and Table  3 also show that the worst prediction under both MT and MT_P scenarios was in environment EHT, whereas the best prediction was in environment Bed2IR. In all environments, MT_P slightly outperformed MT (Figure  4A, WI).

**Table 3:** Dataset 2 EYT 2014–2015

		Models and methods					Models and methods				
		BRR	GBLUP	GK	PK	SK	BRR	GBLUP	GK	PK	SK
			
Env.	Scenario	Without G × E (WI)					With G × E (I)				
DTHD											
Bed2IR	MT	2.66	2.65	**2.40**	2.43	5.90	2.27	2.26	2.05	**2.04**	5.98
Bed5IR	MT	7.68	7.67	**7.21**	7.28	13.23	6.58	6.48	5.66	**5.54**	13.75
EHT	MT	16.13	16.17	**15.55**	15.54	22.87	14.34	14.44	**11.59**	11.76	23.58
Flat5IR	MT	4.34	4.32	**4.03**	4.05	6.34	3.67	**3.62**	3.84	3.79	6.39
LHT	MT	4.34	4.30	4.30	**4.27**	5.25	3.29	3.14	**2.67**	2.87	4.76
Bed2IR	MT_P	2.58	2.61	**2.38**	2.41	5.86	2.22	2.22	**2.01**	2.06	5.92
Bed5IR	MT_P	7.55	7.55	**7.04**	7.15	12.57	6.42	6.37	**5.48**	5.46	12.97
EHT	MT_P	16.19	16.16	**15.50**	15.55	22.72	14.17	14.31	**11.43**	11.74	23.18
Flat5IR	MT_P	4.34	4.33	**4.12**	4.14	6.21	3.69	**3.64**	3.70	3.90	6.23
LHT	MT_P	4.30	4.30	**4.29**	4.32	5.28	3.32	3.15	**2.75**	2.94	4.80
DTMT											
Bed2IR	MT	4.80	4.79	**4.63**	4.70	6.56	4.26	4.20	**3.90**	4.03	6.27
Bed5IR	MT	6.29	6.30	**5.98**	6.05	9.82	5.33	5.36	**4.72**	4.77	10.18
EHT	MT	12.87	12.89	**12.69**	12.75	16.77	11.34	11.44	**9.81**	10.30	17.12
Flat5IR	MT	5.02	4.98	**4.82**	4.87	7.24	4.53	**4.52**	4.65	4.84	7.61
LHT	MT	3.92	3.87	3.90	**3.86**	4.77	3.13	3.05	**2.66**	2.79	4.42
Bed2IR	MT_P	4.68	4.70	**4.52**	4.60	6.54	4.16	4.20	**3.90**	4.07	6.29
Bed5IR	MT_P	5.93	5.95	**5.66**	5.75	8.93	5.07	5.10	**4.51**	4.53	9.19
EHT	MT_P	12.70	12.71	**12.45**	12.55	16.44	11.08	11.22	**9.68**	10.20	16.54
Flat5IR	MT_P	5.05	5.05	**4.90**	4.97	7.06	**4.56**	4.57	4.65	4.95	7.46
LHT	MT_P	3.74	3.71	**3.70**	3.72	4.53	3.01	2.88	**2.59**	2.67	4.26

Average mean squared error (MSE) of prediction for five multitrait multienvironment model-methods: BRR, Bayesian ridge regression; GBLUP, genomic best linear unbiased predictor; GK, Gaussian kernel; PK, polynomial kernel; SK, sigmoidal kernel without G × E (WI) and with G × E (I) for two scenarios (MT and MT_P), four environments (Bed2IR, Bed5IR, EHT, Flat5IR, LHT), and two traits (DTHD, days to heading and DTMT, and days to maturity). Boldface indicates model-method with the lowest MSE for the environment.

**Figure 5:**
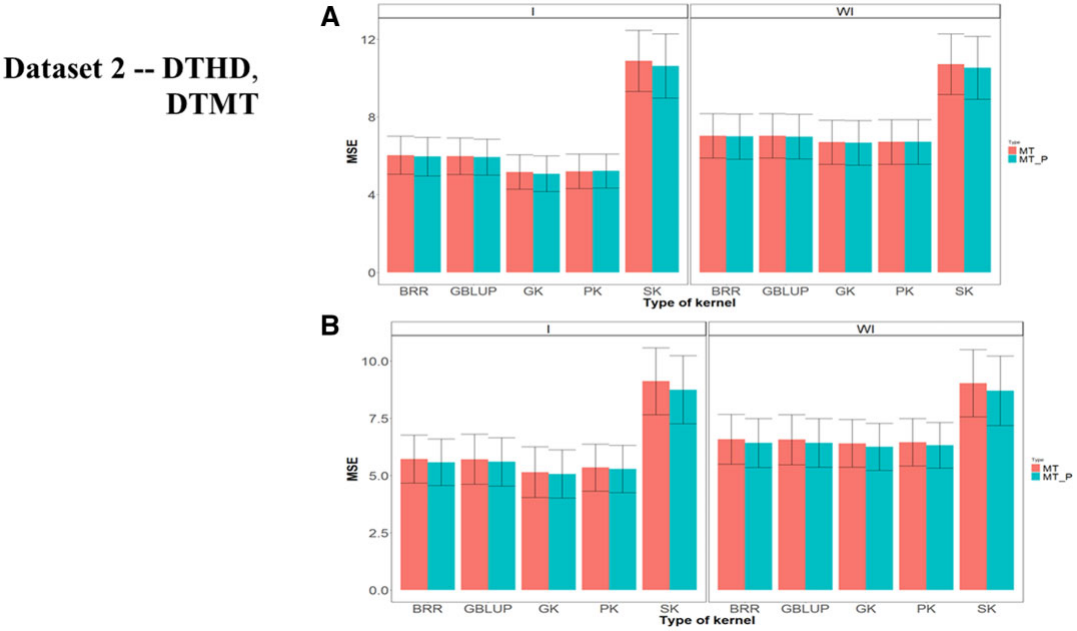
Dataset 2—DTHD and DTMT. Prediction performance across environments in terms of mean square error of prediction (MSE) for traits (A) DTHD with (I) and without (WI) including G × E interaction term for two scenarios (MP and MT_P) and (B) DTMT with (I) and without (WI) including G × E interaction term for two scenarios (MP and MT_P).

Across environments, scenario MT_P slightly outperformed MT (Figure  5A, WI; Table  4). Under MT across environments, the GK kernel performed better than BRR, GBLUP, PK, and SK by 4.96%, 4.86%, 0.258%, and 59.97%, respectively, while for scenario MT_P, the GK outperformed the BRR, GBLUP, PK, and SK by 4.88%, 4.82%, 0.704%, and 57.92%, respectively.

**Table 4:** Dataset 2 EYT 2014–2015.

	Models and methods					Models and methods				
	BRR	GBLUP	GK	PK	SK	BRR	GBLUP	GK	PK	SK
		
Scenario	Without G × E (WI)					With G × E (I)				
DTHD										
MT	7.03	7.02	**6.70**	6.72	10.72	6.03	5.99	**5.16**	5.20	10.89
MT_P	6.99	6.99	**6.67**	6.71	10.53	5.96	5.94	**5.08**	5.22	10.62
DTMT										
MT	6.58	6.57	**6.40**	6.45	9.03	5.72	5.71	**5.15**	5.35	9.12
MT_P	6.42	6.43	**6.25**	6.32	8.70	5.58	5.59	**5.07**	5.29	8.75

Average mean squared error (MSE) prediction, across environments for five model-methods: BRR, Bayesian ridge regression; GBLUP, genomic best linear unbiased predictor; GK, Gaussian kernel; PK, polynomial kernel; SK, sigmoidal kernel without G × E (WI) and with G × E (I) for two scenarios (MT and MT_P) and two traits (DTHD, days to heading and DTMT, days to maturity). Boldface indicates model-method with the lowest MSE for the scenario.

#### DTHD (G × E, I)

When the G × E interaction (Figure  4B, I, and Table  3) term was taken into account for trait DTHD, the best prediction performance under MT occurred under the GK, PK, and GBLUP kernels, but we found differences in the prediction performance of the five methods between environments, since the worst predictions were observed in environment EHT and the best in environment LHT. For this trait, the worst predictions were observed for SK. Under MT_P, the best model was GK (with GBLUP being the best only for Flat5IR).

Sigmoid kernel SK considering the G × E interaction term was also the worst under both scenarios. However, the best performance was observed in environments LHT and EHT under the GK, in environments Bed5IR and Bed2IR with PK and in Flat5IR under GBLUP. No large differences were found in predictions without (Figure  4A) and with (Figure  4B) the G × E interaction term.

Across environments, MT_P was slightly better than the MT scenario (Figure  5A, I, and Table  4). For MT across environments, the GK method had better prediction accuracy than BRR, GBLUP, PK, and SK by 16.67%, 15.95%, 0.716%, and 110.91%, respectively, while for MT_P predictions, the GK method outperformed the BRR, GBLUP, PK and SK by 17.45%, 16.97%, 2.87%, and 109.22%, respectively. As previously found, results including G × E improved the genome-based prediction accuracy as compared to ignoring the interaction term, and MT_P had better prediction accuracy than MT.

#### DTMT (without G × E, WI)

**Figure 6:**
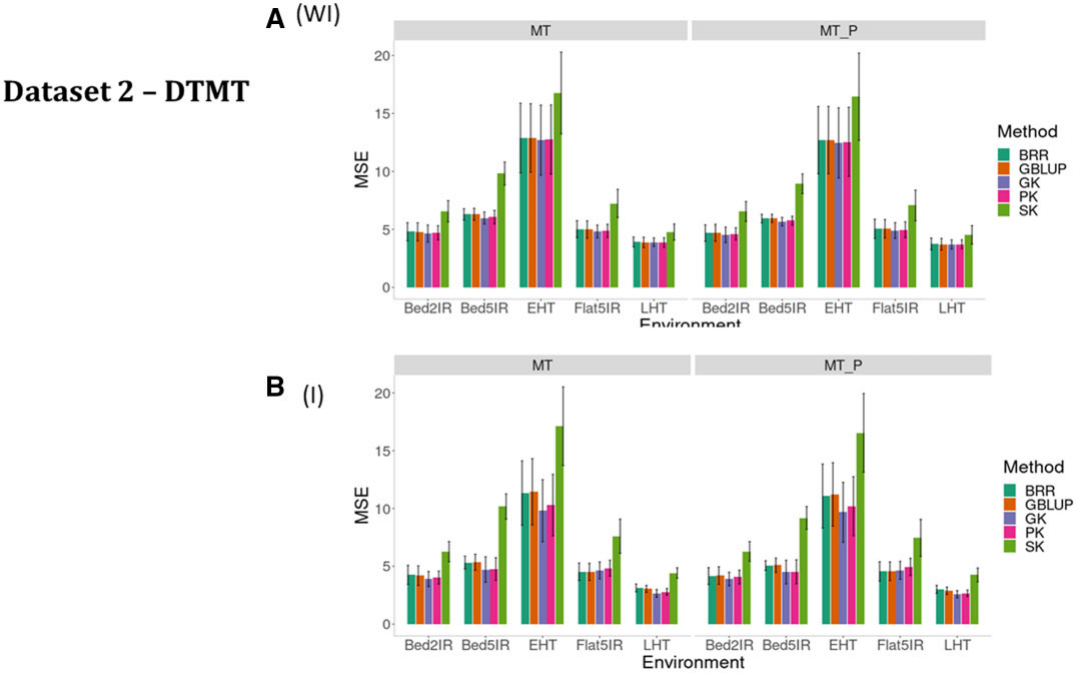
Dataset 2—DTMT. Prediction performance in terms of mean square error of prediction (MSE) for five methods (BRR, GBLUP, GK, PK, and SK) (A) without G × E interaction (WI) and (B) including G × E interaction (I) for five environments (Bed2IR, Bed5IR, EHT, Flat5IR, and LHT) and two scenarios (MT and MT_P).

Figure  6A, WI, and Table  3 show the results of the five methods under both scenarios in terms of MSE without the G × E interaction term for trait DTMT. Results show that the worst performance under both scenarios was observed using the sigmoid kernel (Figure  6A, WI). In general, under MT and MT_P, GK was slightly better than the other four methods. In this trait we found no differences between MT and MT_P (Figure  6A, WI, and Table  3).

Under MT across environments, the GK method outperformed the BRR, GBLUP, PK and SK by 2.72%, 2.53%, 0.69%, and 41.00%, respectively, while under MT_P, the GK method was better than the BRR, GBLUP, PK, and SK by 2.72, 2.82, 1.10 and 39.24%, respectively. In general, the predictions under MT_P were slightly better than those observed under MT (Figure  5B, WI, and Table  4).

#### DTMT (G × E, I)

For trait DTMT, when the G × E interaction (Figure  6B, I, and Table  3) term was taken into account, the best prediction performance under both MT and MT_P was carried out under the GK, but we found differences in the prediction performance of the five methods between environments, since the worst predictions were observed in environment EHT and the best, in environment LHT. For this trait, the worst predictions observed were for SK.

Across environments, scenario MT_P slightly outperformed MT (Figure  5B, I, and Table  4). In all environments, MT_P slightly outperformed MT (Figure  5B, I, and Table  4). Sigmoid kernel SK, taking into account the G × E interaction term, was also the worst under both scenarios. Under scenario MT predictions across environments, the GK was better than BRR, GBLUP, PK, and SK by 11.05%, 10.94%, 3.87%, and 77.14%, respectively, while under MT_P predictions, the GK method overcame the BRR, GBLUP, PK, and SK by 10.07%, 10.44%, 4.35% and 72.65%, respectively (Figure  5B, I, and Table  4).

### Summary of results for dataset 2

Results for dataset 2 were similar to those obtained for dataset 1. The nonlinear multitrait Gaussian kernel had the best genome-based prediction accuracies for most of the environments for both traits (DTHD and DTMT), while the sigmoidal kernel (SK) produced the worse prediction. For the four kernels, the model including G × E and the method (scenario) including MT_P gave better predictions than the model ignoring G × E and/or including all the traits (MT). These patterns are shown in Table  4 and Figure  5.

### Dataset 3 (EYT 2014–2015)

**Figure A1:**
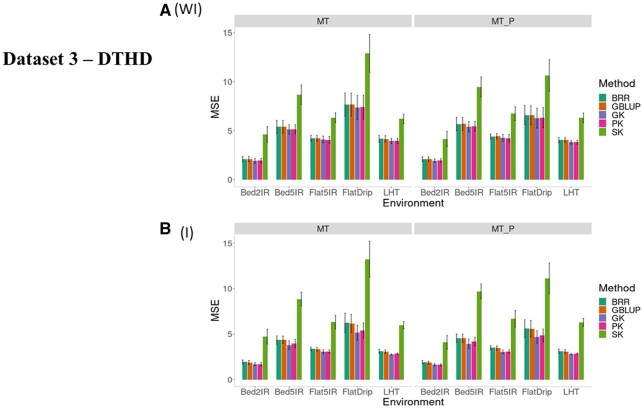
Dataset 3—DTHD. Prediction performance in terms of mean square error of prediction (MSE) for five methods (BRR, GBLUP, GK, PK, and SK) (A) without G × E interaction (WI) and (B) including G × E interaction (I) for five environments (Bed2IR, Bed5IR, EHT, Flat5IR, and LHT) and two scenarios (MT and MT_P).

**Figure A2:**
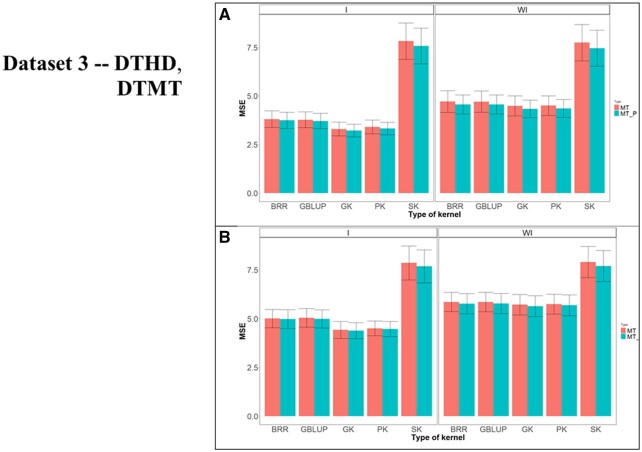
Dataset 3—DTHD and DTMT. Prediction performance across environments in terms of mean square error of prediction (MSE) for traits (A) DTHD with (I) and without (WI) including G × E interaction term for two scenarios (MP and MT_P) and (B) DTMT with (I) and without (WI) including G × E interaction term for two scenarios (MP and MT_P).

**Figure A3:**
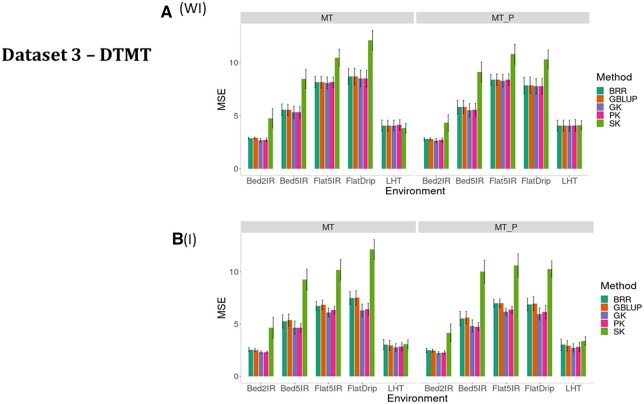
Dataset 3—DTMT. Prediction performance in terms of mean square error of prediction (MSE) for five methods (BRR, GBLUP, GK, PK, and SK) (A) without G × E interaction (WI) and (B) including G × E interaction (I) for five environments (Bed2IR, Bed5IR, EHT, Flat5IR, and LHT) and two scenarios (MT and MT_P).

Details of the results are given in Figures A1A and B, A2A and B, A3A and B and Tables A1 and A2. In dataset 3 under the MT scenario, the models with the G × E interaction outperformed the models that did not include the G × E interaction by 20.30%(BRR), 20.42% (GBLUP), 32.77% (GK), 29.8% (PK), and −0.1% (SK), while under the MT_P, the outperformance was 18.82 (BRR), 19.40 (GBLUP), 31.82 (GK), 29.27 (PK) and −0.6% (SK). In general, the GK was the best genome-based prediction method, together with the model that included the G × E interaction. Further details of the results are given in Appendix A.

## Discussion

### With and without G × E interaction

In general terms, we observed that the best predictions were observed when the G × E interaction term was taken into account, although the superiority with regard to ignoring the G × E interaction went from slight to large. Dataset 1 across environments and traits under the MT scenario with G × E interaction outperformed the models without G × E interaction by 17.51% (BRR), 18.95% (GBLUP), 21.80% (GK), 15.81% (PK), and 1.4% (SK), while under the MT_P scenario, the outperformance of the models with G × E interaction over ignoring the G × E interaction was 14.54% (BRR), 16.47% (GBLUP), 19.30% (GK), 14.91% (PK), and 0.9% (SK). In dataset 2 across environments and traits, the outperformance of the models with the G × E interaction with regard to those that ignored the G × E interaction was 15.83% (BRR), 16.14% (GBLUP), 27.05% (GK), 24.86% (PK), and −1.2% (SK) under scenario MT, while under scenario MT_P, the superiority was 16.20% (BRR), 16.27% (GBLUP), 27.35% (GK), 24.06% (PK), and −0.6% (SK). Finally, in dataset 3 under the MT scenario, the models with the G × E interaction outperformed the models without the G × E interaction by 20.30% (BRR), 20.42% (GBLUP), 32.77% (GK), 29.8% (PK), and −0.1% (SK), while under the MT_P, the outperformance was by 18.82% (BRR), 19.40% (GBLUP), 31.82% (GK), 29.27% (PK), and −0.6% (SK). Note that we only report the results of traits DTHD and DTMT since we did not observe an improvement of the MT model with regard to the UT model for predicting the other two traits (plant height and GY). This could be due to the fact that these two maturity traits (DTHD and DTMT) are highly genetically correlated (with genetic correlations of 0.985, 0.974, and 0.983 in datasets 1, 2, and 3, respectively) and also demonstrate relatively little genotype × environment interaction. Due to the high genetic correlation between these traits, the relative advantage of multivariate approaches will be greater than if traits with lower genetic correlations were used (*e.g.*, plant height and GY). The fact that we did not observe an increase in prediction performance in traits plant height and GY is not rare since this model, as pointed out by one reviewer, should work only for some traits because each trait has a different structure. It is important to point out that our results are in agreement (in terms of the outperformance with regard to no kernel methods) with those obtained in the context of univariate kernel methods (Cuevas *et al.* 2016, 2017, 2018, 2019).

### Under scenarios MT and MT_P

In general terms, we found that the best prediction performance was observed under the MT_P scenario, which was expected, since under this scenario some traits are known and were not predicted. In dataset 1 across environments, traits and type of interaction, the MT_P outperformed the models under the MT scenario by 7.85% (BRR), 7.79% (GBLUP), 7.61% (GK), 7.78% (PK), and 10.76% (SK), while in dataset 2, also across environments, traits and type of interaction, the MT_P scenario outperformed the MT scenario by 1.62% (BRR), 1.37% (GBLUP), 1.54% (GK), 0.73% (PK), and 3.0% (SK). In dataset 3, this outperformance of MT_P over the MT scenario was by 1.74% (BRR), 1.78% (GBLUP), 1.97% (GK), 1.83% (PK), and 2.97% (SK).

### Kernel differences

Under scenarios with and without G × E interaction, the kernel that generally provided the best performance was the GK, which outperformed the other kernels between 0.258% and 110.91%, while the worst performance was observed under the SK kernel. In part these results can be due to a lack of an efficient tuning strategy for the hyperparameters of each kernel. They may also be due to the type of nonlinear patterns of the datasets, the size of the data, and the nature of the kernel function that implements the SK kernel. Also, in general, the GK outperformed the popular GBLUP and BRR models between 2.22% and 17.45%. Even though this superiority is not considerably large, it is a small further step toward improving the GS methodology. We did not apply a significant test to prove that there are significant differences in the performance between the GK and conventional methods (GBLUP and BRR), but we observed the plots. However, since there is overlap of the confidence intervals between the conventional methods (GBLUP and BRR) and GK, we can say that the differences observed only in some cases are significant. In the three datasets evaluated, the GK was always the best genome-based predicted kernel.

### General issues

Kernel methods are powerful tools for the improvement of prediction performance, since they help to capture complex patterns in the data. They also offer flexibility, since they can be implemented in a two-step process using conventional statistical machine learning algorithms, where in the first stage, the kernels are computed, and in the second stage, those kernels are used in conventional linear algorithms. However, although there is empirical evidence that these methods improve the prediction performance in GS under a univariate prediction framework, there are still no generalizations and applications for the multitrait framework. For example, the models/methods used in this study, which when applied to multitrait multienvironment data on the three datasets show consistent improvement in terms of prediction performance mainly with the GK kernel.

Due to the above, in this research we proposed a Bayesian multitrait kernel method to capture nonlinear patterns in the input data under a multitrait framework. The method uses a conventional Bayesian multitrait model that instead of using a linear kernel, allows many types of kernels such as polynomial, Gaussian, sigmoid, etc. Although in the present paper only four kernels were evaluated including the linear kernel, other types of kernels can be considered. This is possible because the implementation of the Bayesian multitrait kernel method is a two-step process in which the kernel is computed in the first stage, and in the second stage the computed kernel replaces the linear kernel of the Bayesian multitrait model. Also, for this reason we do not expect significant differences in the time of implementation between the proposed kernels and the conventional GBLUP model since the number of parameters to estimate between the proposed kernel methods and the GBLUP method are the same.

Our results show that implementing the Bayesian multitrait kernel model improves the prediction performance with regard to the conventional linear multitrait kernel methods, since the Gaussian kernel outperformed conventional methods (Ridge regression and GBLUP) between 5.06% and 10.35% (in dataset 1), between 2.53% and 17.45% (dataset 2) and between 2.22% and 16.39% (dataset 3), and due to the fact that in the three datasets, the proposed method outperformed conventional methods. The proposed method can be implemented with conventional mixed multitrait models because a two-step process is required. It is important to point out that we do not expect the proposed method to outperform the conventional multitrait model in all datasets, since not all datasets are expected to have complex patterns in their input, although in all those datasets with complex nonlinear patterns in the input, the proposed method is expected to be able to improve the prediction performance. The small superiority of the MT model over the UT model could be due, in part, to the small number of markers and not to the strong correlation of the traits. These results, although not strong for improving GS genome-based prediction accuracy, represent a step forward in the right direction.

Another advantage of the Bayesian multitrait kernel methods is that they can significantly reduce the computational resources needed in comparison with Ridge regression multitrait models, since instead of directly using the inputs (independent variables), a transformed input is used that usually has less dimension than the dimension of the number of inputs. However, as with all kernel methods, due to this transformation of the input, the estimates of the beta coefficients are not interpretable as in conventional regression methods, and for this reason, these methods do not help to further understand the complex relationship between input and output, and as such, it is important to avoid false expectations about these methods (Montesinos-López *et al.* 2021) in terms of interpretability. Finally, as one reviewer pointed out, the successful implementation of the multitrait kernel method proposed here is straightforward when the dataset is balanced in the response variable (no missing data) and in the environments, but more complicated when the data are not balanced, but still the method works by only taking care of the imbalance situation. Also, it is important to point out that the phenotypic correlation between environments did not negatively impact the prediction performance of the proposed method since all the phenotypic correlations between environments are positive (Cuevas *et al.* 2016) for all traits (see Appendix C).

Some limitations of the proposed Bayesian multitrait kernel methods are: (1) it is more difficult to tune the hyperparameters of the kernels than in UT kernel methods, (2) that negative phenotypic correlations between environments can negatively affect the prediction performance, as stated by Cuevas *et al.* (2016), and (3) as in UT kernel methods, the beta coefficients resulting from multitrait kernel methods are not interpretable like in conventional linear regression methods, but there is ongoing research to allow variable selection with kernel methods (Crawford *et al.* 2018).

## Conclusions

The proposed Bayesian multitrait kernel method is an attractive and novel approach to capture complex nonlinear patterns in multitrait data that helps take advantage of the correlation between traits. We found that the proposed MT kernel method outperformed the prediction performance of conventional Bayesian multitrait models. However, out of the four nonlinear kernels evaluated, we found that the best performance was obtained using the Gaussian kernel, and the worst, using the sigmoid kernel. In addition, we pointed out that the proposed methods can be implemented in conventional software for Bayesian multitrait models but require a two-step process. In the first step, the kernels are built, and in the second step, those kernels replace the genomic relationship matrices in the multitrait models. Additionally, we provided the data and the R code used in such a way that other scientists can implement this model with their own data.

## Data availability

Phenotypic and genomic data for the three datasets are available at the following link https://hdl.handle.net/11529/10548629.

## Appendix A: Dataset 3 (EYT 2015–2016)

### DTHD (without G × E, WI)

Figure A1A, WI, and Table A1 show the results for the five methods for trait DTHD without including G × E for both scenarios (MT and MT_P). Under both scenarios, we notice that in terms of MSE, the best performance was observed with the Gaussian kernel (GK) and the worst, with the sigmoid kernel (SK). With the exception of the sigmoid kernel (SK), the other four methods were slightly worse than the GK under both scenarios (Figure A1A, WI). For environments FlatDrip, MT outperformed MT_P by a sizeable amount.

Under MT scenario predictions across environments, the GK method outperformed the BRR, GBLUP, PK and SK by 4.95, 4.93%, 0.34%, and 72.68%, respectively, while under MT_P scenario, predictions under the GK method outperformed the BRR, GBLUP, PK and SK by 5.13%, 5.18%, 0.46%, and 72.03%, respectively (Figure A2A, WI, and Table A2). Scenario MT_P gave slightly and consistent increase in prediction accuracy over the MT scenario.

### DTHD (G × E, I)

Considering the model with the G × E interaction term, the sigmoid kernel (SK) was the worst under both MT and MT_P scenarios. The Gaussian kernel (GK) was the best under both scenarios in all environments (Figure A1B, I, and Table A1).

Under MT predictions across environments, the GK method outperformed the BRR, GBLUP, PK and SK by 15.58, 14.60, 3.41 and 137.54%, respectively, while under MT_P predictions, the GK method outperformed the BRR, GBLUP, PK and SK by 16.39, 15.35, 3.25 and 135.61%, respectively (Figure A2A, I, and Table A2). Including the term G × E, the genome-based predictions accuracy increases as shown in Figure A2A, WI and I, and Table A2.

### DTMT (without G × E, WI)

For trait DTMT, Figure A3A, WI, and Table A1 show the results in terms of MSE of five methods under both scenarios. Here, also the sigmoid kernel (SK) was the worst in terms of prediction performance without the interaction term under MT and MT_P. We observed that the GK was slightly better than the other four methods and considerably better than the SK. Between MT and MT_P, only small differences were observed. The worst prediction (Figure A3A, WI) under both scenarios was observed in environment FlatDrip and Flat5IR and the best in environment Bed2IR for M and MT_P.

For the across environment analyses, multitrait GK kernel had the smallest MSE, followed by the PK (Table A2). Slight advantage of the MT_P scenario over the MT was noted (Figure A2B, WI). Under scenario MT predictions across environments, the GK method outperformed the BRR, GBLUP, PK, and SK by 2.41%, 2.33%, 0.48% and 38.25%, respectively, while under MT_P predictions, the GK method outperformed the BRR, GBLUP, PK, and SK by 2.22%, 2.39%, 0.81% and 36.61%, respectively (Figure A2B, WI, and Table A2).

### DTMT (G × E, I)

With the G × E interaction (Figure A3B and Table A1), the best prediction performances were found under the GK for trait DTMT, but we did not find large differences in the prediction performance of the other four methods. However, between environments, we found significant differences and the worst predictions were observed in environment FlatDrip and the best in environment Bed2IR. In all methods and scenarios, the worst predictions were observed under the SK method.

Also, the predictions under MT_P across environments were slightly better than under MT Table A2 and clearly superior in environment FlatDrip (Figure A3B). Under scenario MT predictions across environments, the GK method outperformed the BRR, GBLUP, PK, and SK by 13.27%, 13.97%, 1.85%, and 77.75%, respectively, while under MT_P predictions, the GK method also outperformed the BRR, GBLUP, PK, and SK by 13.67%, 13.84%, 1.98%, and 75.72%, respectively (Figure A2B, I, and Table A2). Finally, differences were observed between the predictions without (Figure A3A, WI) and with (Figure A3B, I) the G × E interaction term.

**Table A1:** Dataset 3 EYT 2015–2016

		Models and methods					Models and methods				
		BRR	GBLUP	GK	PK	SK	BRR	GBLUP	GK	PK	SK
			
Env.	Scenario	Without G × E (WI)					With G × E (I)				
DTHD											
Bed2IR	MT	2.09	2.10	**1.90**	1.93	4.61	1.94	1.89	**1.71**	1.71	4.73
Bed5IR	MT	5.39	5.40	**5.12**	5.15	8.69	4.35	4.38	**3.77**	3.98	8.86
Flat5IR	MT	4.22	4.22	4.10	**4.07**	6.33	3.37	3.36	**3.06**	3.09	6.31
FlatDrip	MT	7.67	7.66	**7.36**	7.39	12.90	6.24	6.17	**5.16**	5.39	13.23
LHT	MT	4.16	4.15	**3.94**	3.96	6.21	3.12	3.08	**2.77**	2.85	5.99
Bed2IR	MT_P	2.08	2.08	**1.93**	1.97	4.15	1.92	1.86	**1.64**	1.64	4.10
Bed5IR	MT_P	5.68	5.68	**5.40**	5.43	9.47	4.55	4.55	**3.93**	4.18	9.70
Flat5IR	MT_P	4.41	4.42	4.25	**4.22**	6.73	3.50	3.46	**3.05**	3.07	6.70
FlatDrip	MT_P	6.58	6.57	**6.28**	6.33	10.64	5.61	5.59	**4.67**	4.85	11.11
LHT	MT_P	4.04	4.04	**3.82**	3.83	6.31	3.12	3.08	**2.79**	2.87	6.28
DTMT											
Bed2IR	MT	2.87	2.88	**2.67**	2.71	4.74	2.54	2.51	**2.31**	**2.31**	4.65
Bed5IR	MT	5.54	5.53	**5.30**	5.30	8.46	5.24	5.37	4.65	**4.63**	9.26
Flat5IR	MT	8.15	8.13	**8.07**	8.14	10.44	6.72	6.83	**6.11**	6.32	10.19
FlatDrip	MT	8.67	8.66	8.49	**8.47**	12.08	7.49	7.52	**6.28**	6.42	12.13
LHT	MT	**4.05**	**4.05**	4.06	4.11	3.81	3.03	2.96	**2.75**	2.83	3.06
Bed2IR	MT_P	2.80	2.80	**2.65**	2.69	4.31	2.47	2.45	**2.23**	2.27	4.16
Bed5IR	MT_P	5.79	5.80	**5.50**	5.54	9.09	5.52	5.60	4.81	**4.74**	10.02
Flat5IR	MT_P	8.37	8.39	**8.27**	8.37	10.77	7.00	6.99	**6.18**	6.38	10.60
FlatDrip	MT_P	7.83	7.85	**7.74**	7.76	10.27	6.87	6.95	**5.96**	6.13	10.27
LHT	MT_P	*4.03*	4.04	**4.03**	4.06	4.09	3.02	2.93	**2.71**	2.80	3.37

Average mean squared error (MSE) of prediction for five multitrait multi-environment model-methods: BRR, Bayesian ridge regression; GBLUP, genomic best linear unbiased predictor; GK, Gaussian kernel; PK, polynomial kernel; SK, sigmoidal kernel without G × E (WI) and with G × E (I) for two scenarios (MT and MT_P) for five environments (Bed 2IR, Bed5IR, EHT, Flat5IR, and LHT) and two traits (DTHD, days to heading and DTMT, days to maturity). Boldface indicates model method with the lowest MSE for the environment.

**Table A2:** Dataset 3 EYT 2014–2015

	Models and methods					Models and methods				
	BRR	GBLUP	GK	PK	SK	BRR	GBLUP	GK	PK	SK
		
Scenario	Without G × E (WI)					With G × E (I)				
DTHD										
MT	4.71	4.71	**4.49**	4.50	7.75	3.81	3.77	**3.29**	3.41	7.82
MT_P	4.56	4.56	**4.34**	4.36	7.46	3.74	3.71	**3.22**	3.32	7.58
DTMT										
MT	5.85	5.85	**5.72**	5.74	7.90	5.01	5.04	**4.42**	4.50	7.86
MT_P	5.77	5.77	**5.64**	5.69	7.70	4.98	4.99	**4.38**	4.47	7.69

Average mean squared error (MSE) prediction, across environments for five model-methods: BRR, Bayesian ridge regression; GBLUP, genomic best linear unbiased predictor; GK, Gaussian kernel; PK, polynomial kernel; SK, sigmoidal kernel without G × E (WI) and with G × E (I) for two scenarios (MT and MT_P) and two traits (DTHD, days to heading and DTMT, days to maturity). Boldface indicates model-method with the lowest MSE for the scenario.

## Appendix B: R code

### R code for computing the four kernels

**#########Gaussian Kernel##################**

l2norm=function(x){sqrt(sum(x ^2))}

K.radial=function(x1, x2 = x1, gamma = 1){

exp(-gamma*outer(1: nrow(x1 <- as.matrix(x1)), 1: ncol(x2 <- t(x2)),

Vectorize(function(i, j) l2norm(x1[i , ]-x2[, j]) ^2)))}

KK=K.radial(x1 = **X**, x2 = **X**, gamma = 1) #### **X** is the scaled marker matrix divided by the square ##root of the total number of markers.

**#########Polynomial Kernel##################**

K.polynomial=function(x1, x2 = x1, gamma = 1, b = 0, p = 3) {

(gamma*(as.matrix(x1)%*%t(x2))+b)^p}

KK=K.polynomial(x1= **X**, x2= **X**, gamma = 1) #### **X** is the scaled marker matrix divided by the ###square root of the total number of markers.

**#########Sigmoid Kernel##################**

K.sigmoid=function(x1, x2 = x1, gamma = 1, b = 0)

{tanh(gamma*(as.matrix(x1)%*%t(x2))+b)}

KK=K.sigmoid(x1= **X**, x2= **X**, gamma = 1) #### **X** is the scaled marker matrix divided by the square ##root of the total number of markers.

**#########Linear Kernel##################**

K.linear=function(x1, x2 = x1, gamma = 1) {gamma*(as.matrix(x1)%*%t(as.matrix(x2)))}

KK=K.linear(x1= **X**, x2= **X**, gamma = 1) #### **X** is the scaled marker matrix divided by the square ##root of the total number of markers.

### Implementation of the models using BGLR

load('Pheno.RData',verbose=TRUE) ### Pheno contains at least ###four columns Lines, Environment (Env) and at least ##to columns of the response variables (**Y**).

#########Compute the design matrix of lines and environments

nt= ncol(Pheno)-2 ####number of traits under study

XE = model.matrix(∼0+as.factor(Env),data=Pheno)

KE= XE%*%t(XE)

ZL = model.matrix(∼0+as.factor(Line),data=Pheno)

KL=ZL%*%KK%*%t(ZL)

########Interaction GxE###########

KLE=KE*KL

**ETA** = list(Env=list (X=XE[,-1], model=**‘FIXED’)**, Line=list (K=KL, model=‘**RKHS**’), LinexEnv= list (K=KLE model=’ **RKHS**’))

A = Multitrait(y = **Y**, ETA=**ETA**, resCov = list (type = ‘**UN**’, S0 = diag(nt), df0 = 5),

nIter = **10,000**, burnIn = **2000**)

## Appendix C: Phenotypic correlations of the three datasets

**Table C1:** Phenotypic correlation of dataset 1

Trait	Env	Bed5IR	EHT	Flat5IR	LHT
DTHD	Bed5IR	1.000	0.805	0.846	0.829
DTHD	EHT	0.805	1.000	0.701	0.830
DTHD	Flat5IR	0.846	0.701	1.000	0.712
DTHD	LHT	0.829	0.830	0.712	1.000
DTMT	Bed5IR1	1.000	0.767	0.731	0.761
DTMT	EHT1	0.767	1.000	0.695	0.758
DTMT	Flat5IR1	0.731	0.695	1.000	0.577
DTMT	LHT1	0.761	0.758	0.577	1.000
GY	Bed5IR2	1.000	0.392	0.344	0.138
GY	EHT2	0.392	1.000	0.248	0.002
GY	Flat5IR2	0.344	0.248	1.000	0.035
GY	LHT2	0.138	0.002	0.035	1.000
Height	Bed5IR3	1.000	0.516	0.518	0.323
Height	EHT3	0.516	1.000	0.386	0.261
Height	Flat5IR3	0.518	0.386	1.000	0.420
Height	LHT3	0.323	0.261	0.420	1.000

**Table C2:** Phenotypic correlation of dataset 2

Trait	Env	Bed2IR	Bed5IR	EHT	Flat5IR	LHT
DTHD	Bed2IR	1.000	0.876	0.821	0.805	0.849
DTHD	Bed5IR	0.876	1.000	0.732	0.877	0.768
DTHD	EHT	0.821	0.732	1.000	0.718	0.776
DTHD	Flat5IR	0.805	0.877	0.718	1.000	0.699
DTHD	LHT	0.849	0.768	0.776	0.699	1.000
DTMT	Bed2IR1	1.000	0.760	0.649	0.650	0.724
DTMT	Bed5IR1	0.760	1.000	0.675	0.842	0.742
DTMT	EHT1	0.649	0.675	1.000	0.646	0.693
DTMT	Flat5IR1	0.650	0.842	0.646	1.000	0.656
DTMT	LHT1	0.724	0.742	0.693	0.656	1.000
GY	Bed2IR2	1.000	0.425	0.313	0.347	0.193
GY	Bed5IR2	0.425	1.000	0.456	0.618	0.293
GY	EHT2	0.313	0.456	1.000	0.410	0.298
GY	Flat5IR2	0.347	0.618	0.410	1.000	0.238
GY	LHT2	0.193	0.293	0.298	0.238	1.000
Height	Bed2IR3	1.000	0.381	0.406	0.450	0.328
Height	Bed5IR3	0.381	1.000	0.339	0.502	0.290
Height	EHT3	0.406	0.339	1.000	0.470	0.445
Height	Flat5IR3	0.450	0.502	0.470	1.000	0.423
Height	LHT3	0.328	0.290	0.445	0.423	1.000

**Table C3:** Phenotypic correlation of dataset 3

Trait	Env	Bed2IR	Bed5IR	Flat5IR	FlatDrip	LHT
DTHD	Bed2IR	1.000	0.786	0.762	0.871	0.771
DTHD	Bed5IR	0.786	1.000	0.726	0.716	0.693
DTHD	Flat5IR	0.762	0.726	1.000	0.728	0.628
DTHD	FlatDrip	0.871	0.716	0.728	1.000	0.754
DTHD	LHT	0.771	0.693	0.628	0.754	1.000
DTMT	Bed2IR1	1.000	0.717	0.560	0.725	0.642
DTMT	Bed5IR1	0.717	1.000	0.635	0.642	0.563
DTMT	Flat5IR1	0.560	0.635	1.000	0.498	0.437
DTMT	FlatDrip1	0.725	0.642	0.498	1.000	0.541
DTMT	LHT1	0.642	0.563	0.437	0.541	1.000
GY	Bed2IR2	1.000	0.232	0.121	0.608	0.125
GY	Bed5IR2	0.232	1.000	0.250	0.092	0.361
GY	Flat5IR2	0.121	0.250	1.000	0.117	0.025
GY	FlatDrip2	0.608	0.092	0.117	1.000	0.002
GY	LHT2	0.125	0.361	0.025	0.002	1.000
Height	Bed2IR3	1.000	0.367	0.407	0.160	0.258
Height	Bed5IR3	0.367	1.000	0.381	0.054	0.360
Height	Flat5IR3	0.407	0.381	1.000	0.319	0.229
Height	FlatDrip3	0.160	0.054	0.319	1.000	0.118
Height	LHT3	0.258	0.360	0.229	0.118	1.000

## References

[jkab406-B1] Arojju SK , Cao M, Trolove M, Barrett BA, Inch C, et al 2020. Multi-trait genomic prediction improves predictive ability for dry matter yield and water-soluble carbohydrates in perennial ryegrass. Front Plant Sci. 11:1197.doi: 10.3389/fpls.2020.01197 32849742 10.3389/fpls.2020.01197 PMC7426495

[jkab406-B2] Bradbury PJ , Zhang Z, Kroon DE, Casstevens TM, Ramdoss Y, et al 2007. TASSEL: software for association mapping of complex traits in diverse samples. Bioinformatics. 23:2633–2635. doi: 10.1093/bioinformatics/btm308. 17586829 10.1093/bioinformatics/btm308

[jkab406-B3] Calus MP , Veerkamp RF. 2011. Accuracy of multi-trait genomic selection using different methods. Genet Sel Evol. 43:26.doi: 10.1186/1297–9686-43-26. 21729282 10.1186/1297-9686-43-26 PMC3146811

[jkab406-B4] Crossa J , de los Campos G, Pérez P, Gianola D, Burgueño J, et al 2010. Prediction of genetic values of quantitative traits in plant breeding using pedigree and molecular markers. Genetics. 186:713–724. doi: 10.1534/genetics.110.118521. 20813882 10.1534/genetics.110.118521 PMC2954475

[jkab406-B5] Cuevas J , Crossa J, Soberanis V, Pérez-Elizalde S, Pérez-Rodríguez P, et al 2016. Genomic prediction of genotype × environment interaction kernel regression models. Plant Genome. 9:1:20. 10.3835/plantgenome2016.03.0024 27902799

[jkab406-B6] Cuevas J , Crossa J, Montesinos-López OA, Burgueño J, Pérez-Rodríguez P, et al 2017. Bayesian genomic prediction with genotype × environment kernel models. G3 (Bethesda). 7:41–53. 27793970 10.1534/g3.116.035584 PMC5217122

[jkab406-B7] Cuevas J , Granato I, Fritsche-Neto R, Montesinos-Lopez OA, Burgueño J, et al 2018. Genomic-enabled prediction kernel models with random intercepts for multi-environment trials. G3 (Bethesda). 8:1347–1365. 29476023 10.1534/g3.117.300454 PMC5873923

[jkab406-B8] Cuevas J , Montesinos-López OA, Juliana P, Guzmán C, Pérez-Rodríguez P, et al 2019. Deep kernel for genomic and near infrared predictions in multi-environment breeding trials. G3 (Bethesda). 9:2913–2924. 31289023 10.1534/g3.119.400493 PMC6723142

[jkab406-B9] Cuevas J , Montesinos-López OA, Martini JWR, Pérez-Rodríguez P, Lillemo M, et al 2020. Approximate genome-based kernel models for large datasets including main effects and interactions. Front Genet. 11:567757. doi: 10.3389/fgene.2020.567757. 33193659 10.3389/fgene.2020.567757 PMC7594507

[jkab406-B10] Crawford L , Wood KC, Zhou X, Mukherjee S. 2018. Bayesian approximate kernel regression with variable selection. J Am Stat Assoc. 113:1710–1721. doi:10.1080/01621459.2017.1361830. 30799887 10.1080/01621459.2017.1361830 PMC6383716

[jkab406-B11] de los Campos G , Pérez-Rodríguez P. 2014. Bayesian Generalized Linear Regression. R package version 1.0.4. http://CRAN.R-project.org/package=BGLR.

[jkab406-B12] Ducrocq V. 1994. Multiple trait prediction: principles and problems. In: Proceedings of the 5th world congress on genetics applied to livestock production, 7–12 August 1994, Guelph.

[jkab406-B13] Elshire RJ , Glaubitz JC, Sun Q, Poland JA, Kawamoto K, et al 2011. A robust, simple genotyping-by-sequencing (GBS) approach for high diversity species. PLoS One. 6:e19379.doi: 10.1371/journal.pone.0019379. 21573248 10.1371/journal.pone.0019379 PMC3087801

[jkab406-B14] Falconer DS , Mackay TFC. 1996. Introduction to quantitative genetics. 4th ed. Harlow: Addison Wesley Longman.

[jkab406-B15] Gianola D , van Kaam JBCHM. 2008. Reproducing kernel Hilbert spaces regression methods for genomic assisted prediction of quantitative traits. Genetics. 178:2289–2303. doi: 10.1534/genetics.107. 084285. 18430950 10.1534/genetics.107.084285 PMC2323816

[jkab406-B16] He D , Kuhn D, Parida L. 2016. Novel applications of multitask learning and multiple output regression to multiple genetic trait prediction. Bioinformatics. 32:i37–i43. doi: 10.1093/bioinformatics/btw249. 27307640 10.1093/bioinformatics/btw249 PMC4908333

[jkab406-B17] Jarquín D , Crossa J, Lacaze X, Du Cheyron P, Daucourt J, et al 2014. A reaction norm model for genomic selection using high-dimensional genomic and environmental data. Theor Appl Genet. 127:595–607. 24337101 10.1007/s00122-013-2243-1 PMC3931944

[jkab406-B18] Jia Y , Jannink J-L. 2012. Multiple-trait genomic selection methods increase genetic value prediction accuracy. Genetics. 192:1513–1522. doi: 10.1534/genetics.112.144246. 23086217 10.1534/genetics.112.144246 PMC3512156

[jkab406-B19] Jiang J , Zhang Q, Ma L, Li J, Wang Z, et al 2015. Joint prediction of multiple quantitative traits using a Bayesian multivariate antedependence model. Heredity (Edinb). 115:29–36. 25873147 10.1038/hdy.2015.9 PMC4815501

[jkab406-B20] Juliana P. J , Singh R. P, Poland J, Mondal S, Crossa et al 2018. Prospects and challenges of applied genomic selection-a new paradigm in breeding for grain yield in bread wheat. Plant Genome. 11:180017.doi:10.3835/plantgenome218.03.0017. 10.3835/plantgenome2018.03.0017 PMC7822054 30512048

[jkab406-B21] Long N , Gianola D, Rosa GJ, Weigel KA, Kranis A, et al 2010. Radial basis function regression methods for predicting quantitative traits using SNP markers. Genet Res (Camb). 92:209–225. doi: 10.1017/S0016672310000157. 20667165 10.1017/S0016672310000157

[jkab406-B22] Mbebi AJ , Tong H, Nikoloski Z. 2021. L_2,1_-norm regularized multivariate regression model with applications to genomic prediction. Bioinformatics. 37:2896–2904. doi: 10.1093/bioinformatics/btab212. 10.1093/bioinformatics/btab212 PMC8479665 33774677

[jkab406-B23] Meuwissen THE , Hayes BJ, Goddard ME. 2001. Prediction of total genetic value using genome wide dense marker maps. Genetics. 157:1819–1829. 11290733 10.1093/genetics/157.4.1819 PMC1461589

[jkab406-B24] Money D , Gardner K, Migicovsky Z, Schwaninger H, Zhong G, et al 2015. LinkImpute: fast and accurate genotype imputation for nonmodel organisms. G3 (Bethesda). 5:2383–2390. doi: 10.1534/g3.115.021667. 26377960 10.1534/g3.115.021667 PMC4632058

[jkab406-B25] Montesinos-López OA , Montesinos-López A, Crossa J, Toledo F, Pérez-Hernández O, et al 2016. A genomic Bayesian multi-trait and multi-environment model. G3 (Bethesda). 6:2725–2744. 27342738 10.1534/g3.116.032359 PMC5015931

[jkab406-B27] Montesinos-López OA , Montesinos-López A, Crossa J, Gianola D, Hernández-Suárez CM, et al 2018. Multi-trait, multi-environment deep learning modeling for genomic-enabled prediction of plant traits. G3: genes|Genomes|Genetics. G3 (Bethesda). 8:3829–3840. doi:10.1534/g3.118.200728. 30291108 10.1534/g3.118.200728 PMC6288830

[jkab406-B26] Montesinos-López OA , Montesinos-López A, Crossa J, Kismiantini Ramírez-Alcaraz JM, Singh R, et al 2019a. A singular value decomposition Bayesian multiple-trait and multiple-environment genomic model. Heredity (Edinb). 122: 381–401. 10.1038/s41437-018-0109-7. 30120367 PMC6460759

[jkab406-B28] Montesinos-López OA , Montesinos-López A, Crossa J, Cuevas J, Montesinos-López JC, Salas-Gutiérrez, et al 2019b. A Bayesian genomic multi-output regressor stacking model for predicting multi-trait multi-environment plant breeding data. G3 (Bethesda). 3381–3393. 31427455 10.1534/g3.119.400336 PMC6778812

[jkab406-B29] Montesinos-López OA , Martín-Vallejo J, Crossa J, Gianola D, Hernández-Suárez CM, Montesinos-López, et al 2019c. New deep learning genomic prediction model for multi-traits with mixed binary, ordinal, and continuous phenotypes. G3 (Bethesda). 9:1545–1556. 30858235 10.1534/g3.119.300585 PMC6505163

[jkab406-B30] Montesinos-López OA , Montesinos-López JC, Singh P, Lozano-Ramirez N, Barrón-López A, et al 2020. A multivariate Poisson deep learning model for genomic prediction of count. G3 (Bethesda). 10:4177–4190. 32934019 10.1534/g3.120.401631 PMC7642922

[jkab406-B31] Montesinos-López A , Montesinos-López OA, Montesinos-López JC, Flores-Cortes CF, de la Rosa R, et al 2021. A guide for kernel generalized regression methods for genomic-enabled prediction, heredity. doi: 10.1038/s41437-021–00412-1. 10.1038/s41437-021-00412-1 PMC8115678 33649571

[jkab406-B32] Neyhart JL , Lorenz AJ, Smith KP. 2019. Multi-trait improvement by predicting genetic correlations in breeding crosses. G3 (Bethesda). 9:3153–3165. doi: 10.1534/g3.119.400406. 31358561 10.1534/g3.119.400406 PMC6778794

[jkab406-B33] Okeke UG , Akdemir D, Rabbi I, Kulakow P, Jannink JL. 2017. Accuracies of univariate and multivariate genomic prediction models in African cassava. Genet Sel Evol. BioMed Central. 49:1–10. 10.1186/s12711-017-0361-y PMC5715664 29202685

[jkab406-B34] Poland JA , Brown PJ, Sorrells ME, Jannink JL. 2012. Development of high-density genetic maps for barley and wheat using a novel two-enzyme genotyping-by-sequencing approach. PLoS One. 7:e32253.doi: 10.1371/journal.pone.0032253. 22389690 10.1371/journal.pone.0032253 PMC3289635

[jkab406-B35] R Core Team. 2020. R: A Language and Environment for Statistical Computing. R Foundation for Statistical Computing, Vienna, Austria. ISBN 3–900051-07-0. URL http://www.R-project.org/.

[jkab406-B36] Runcie DE , Qu J, Cheng H, Crawford L. 2021. MegaLMM: mega-scale linear mixed models for genomic predictions with thousands of traits. Genome Biol. 22:213.doi: 10.1186/s13059-021–02416-w. 34301310 10.1186/s13059-021-02416-w PMC8299638

[jkab406-B37] Schulthess AW , Zhao Y, Longin CFH, Reif JC. 2018. Advantages and limitations of multiple-trait genomic prediction for Fusarium head blight severity in hybrid wheat. Theor Appl Genet. 131:685–701. doi: 10.1007/s00122-017–3029-7. 29198016 10.1007/s00122-017-3029-7

[jkab406-B38] Shawe-Taylor J , Cristianini N. 2004. Kernel Methods for Pattern Analysis. Cambridge, UK: University Press.

[jkab406-B39] Tong H , Küken A, Nikoloski Z. 2020. Integrating molecular markers into metabolic models improves genomic selection for *Arabidopsis* growth. Nat Commun. 11:2410.doi: 10.1038/s41467-020–16279-5 32415110 10.1038/s41467-020-16279-5 PMC7229213

[jkab406-B40] Van Raden PM. 2008. Efficient method to compute genomic predictions. J Dairy Sci. 91:4414–4423. 18946147 10.3168/jds.2007-0980

[jkab406-B41] van der Werf J , van Arendonk JAM, De Vries AG. 1992. Improving selection of pigs using correlated characters. In: Proceedings of the 43rd Annual Meeting of the European Federation of Animal Science (EAAP), 14–17 September 1992, Madrid. 1992. p. 18.

[jkab406-B42] Zhou X , Stephens M. 2014. Efficient multivariate linear mixed model algorithms for genome-wide association studies. Nat Methods. 11:407–409. 24531419 10.1038/nmeth.2848 PMC4211878

